# Electroencephalographic Correlates and Predictors of Treatment Outcome in OCD: A Brief Narrative Review

**DOI:** 10.3389/fpsyt.2021.703398

**Published:** 2021-08-02

**Authors:** Brian A. Zaboski, Elisa F. Stern, Patrick D. Skosnik, Christopher Pittenger

**Affiliations:** Department of Psychiatry, Yale School of Medicine, Yale University, New Haven, CT, United States

**Keywords:** electroencaphlography, EEG, obsessive-compulsive disorder, biomarker, brain imaging correlates, predictors

## Abstract

Electroencephalography (EEG) measures the brain's electrical activity with high temporal resolution. In comparison to neuroimaging modalities such as MRI or PET, EEG is relatively cheap, non-invasive, portable, and simple to administer, making it an attractive tool for clinical deployment. Despite this, studies utilizing EEG to investigate obsessive-compulsive disorder (OCD) are relatively sparse. This contrasts with a robust literature using other brain imaging methodologies. The present review examines studies that have used EEG to examine predictors and correlates of response in OCD and draws tentative conclusions that may guide much needed future work. Key findings include a limited literature base; few studies have attempted to predict clinical change from EEG signals, and they are confounded by the effects of both pharmacotherapy and psychotherapy. The most robust literature, consisting of several studies, has examined event-related potentials, including the P300, which several studies have reported to be abnormal at baseline in OCD and to normalize with treatment; but even here the literature is quite heterogeneous, and more work is needed. With more robust research, we suggest that the relatively low cost and convenience of EEG, especially in comparison to fMRI and PET, make it well-suited to the development of feasible personalized treatment algorithms.

Obsessive-compulsive disorder (OCD) is defined by clinically significant obsessions and/or compulsions. Obsessions are unwanted, intrusive thoughts that cause distress and are unrealistic or excessive. Compulsions are repetitive behaviors that neutralize anxiety or distress caused by obsessions (1). Estimates of lifetime prevalence range from 1 to 4% of adults; the attendant disability is substantial (2–5). Unfortunately, precision medicine—establishing who may benefit most from existing treatments—remains a distant goal. Efforts in this direction have begun to incorporate neuroscientific methodologies, including electroencephalography (EEG). The present review seeks to summarize the limited body of literature focused on the EEG correlates and predictors of treatment response in OCD.

Diagnosis and assessment of OCD depend on clinical interviews and rating scales that quantify symptoms and identify functional impairments (6), such as the Yale-Brown Obsessive-Compulsive Scale (YBOCS) (7). It would be useful to complement, validate, and refine this descriptive clinical nosology with objective biomarkers (8). As such, the search for biological correlates has been a major thrust of research since the 1980s. Toward this end, early PET and fMRI studies identified hypermetabolism in cortico-striatal-thalamo-cortical circuitry, particularly in the orbito-frontal cortex, anterior cingulate cortex, and caudate nucleus (9). Large structural neuroimaging studies have described various abnormalities in OCD patients, including increased globus pallidus volume, reduced cortical thickness in the inferior parietal cortex, and lower surface area of the transverse temporal cortex (10). However, small effect sizes of these functional and anatomical abnormalities prevent clinically actionable practices, and even if more robust findings were identified, these imaging and analytic methodologies are impractical in most clinical settings.

First-line treatment for OCD [e.g., (11)] includes exposure-based cognitive-behavioral therapy (CBT), an intervention that assists clients to approach fear-inducing stimuli and build new neural connections that inhibit fear (6, 12). If after receiving CBT for a reasonable duration (12–16 sessions) a patient does not experience adequate symptom alleviation, therapy can be augmented or replaced by a pharmacological intervention, typically a selective serotonin reuptake inhibitor (SSRI) (11). Meta-analyses demonstrate benefits with large effect sizes for both treatment modalities (13, 14).

Many individuals with OCD do not respond to existing treatments, so numerous studies over the past decade have sought to characterize the neural changes that predict or accompany symptom improvement during treatment. Importantly, treatment predictors and correlates may be distinct. Correlation indicates that two variables—like a measure of brain function and a measure of symptom improvement—are associated; these relationships can be established retrospectively and do not satisfy claims of causation. In contrast, prediction suggests that a variable, such as a pre-treatment measure of brain activity, can anticipate the subsequent value of another, such as treatment response. The study designs and statistical analyses required to develop predictive claims are distinct from those required to establish correlation (15, 16).

Positron emission tomography (PET) imaging of brain perfusion and metabolic activity has been used to examine treatment correlates since the 1990s. One meta-analysis compiled 14 studies that treated patients with pharmacotherapy (SSRI or clomipramine) or CBT and measured cerebral blood flow or glucose metabolism (17). Across these studies, metabolic activity in the caudate, orbitofrontal cortex, and thalamus declined by the end of treatment, though average effect sizes were small. Recent studies using fMRI have built upon this research, filling important gaps in the literature by employing predictive frameworks. For example, in a randomized treatment trial, researchers found that baseline activation in the right temporal lobes and rostral anterior cingulate cortex during cognitive control, and in ventromedial prefrontal, orbitofrontal, lateral prefrontal cortex, and amygdala during reward processing, were associated with better CBT response (18).

By contrast to the substantial PET and fMRI literature, few studies have used electroencephalography (EEG) to characterize OCD treatment predictors and correlates. This is unfortunate, as EEG has both practical and scientific strengths. EEG is cheaper and easier to acquire than PET and fMRI and is therefore more easily deployed in clinical practice. EEG non-invasively measures electric fields generated by neural activity using scalp electrodes with high temporal resolution (19). As such, EEG is sensitive to neural synchronization and periodicity at time-scales commensurate with real-world perceptual and cognitive processing. These oscillatory signals can be quantified in different frequency bands, typically labeled by increasing frequency: delta (0.5–3 Hz), theta (4–7 Hz), alpha (8–12 Hz), beta (13–29 Hz), and gamma (30–100 Hz). EEG's temporal resolution is excellent, measured in milliseconds—compared to seconds in fMRI studies (20, 21). Despite its poor spatial resolution relative to fMRI and PET—especially for structures deep in the brain—aberrant EEG patterns have contributed to an understanding of numerous neuropsychiatric disorders, including panic disorder, post-traumatic stress disorder, autism, and anxiety disorders (22). In OCD, a recent systematic review described frontal asymmetries in alpha and theta bands, increased error related negativity, and perturbed REM sleep (23).

We provide a brief narrative review of the small EEG literature applied to the study of predictors and correlates of OCD treatment outcomes. Articles were located through PubMed, ProQuest, and Google Scholar and spanned all years. Included studies were treatment studies that included EEG predictors and/or correlates for OCD symptomatology. Developmentally focused studies including pediatric populations were excluded. As more work is needed in this area, we conclude with future research directions. If robust EEG predictors of treatment response can be identified, this approach may make it a valuable tool for biomarker-guided treatment selection and a move toward a precision medicine approach in the treatment of OCD.

## Error-Related Potentials

OCD is characterized by excessive doubt, worry, and intolerance of uncertainty (24), which are reflected by abnormalities in error monitoring and response inhibition (25). When subjects make an error, correlates are observed in a fronto-central event-related potential (ERP), a time-locked pattern of brain activity (26). One ERP component that may differentiate symptom severity and treatment response in OCD is error-related negativity (ERN).

ERNs are observed following behavioral errors or failures of response inhibition, typically during go/no go or flanker tasks (27). The ERN is a negative ERP component that peaks 80–150 ms after the beginning of an erroneous response (28). The CRN is the corresponding response, typically of lower amplitude, after a correct response (29). These can emerge regardless of whether the participant is consciously aware of their error, suggesting that they are capturing subconscious or preconscious processes.

Riesel et al. (30) examined the ERN and CRN using the common Flanker Task (31). In this task, participants are shown stimuli with patterns that are congruent (a row of arrows pointing in the same direction), incongruent (a row of arrows, all but one pointing in the same direction), or neutral (one arrow presented). Participants must then rapidly indicate which they see. Incongruent trials are more difficult and often lead to errors. Pre-treatment, participants with OCD showed larger amplitudes in the ERN and CRN compared to healthy controls. These larger amplitudes persisted following psychotherapy despite symptom improvement. The researchers concluded that ERN abnormalities may represent an OCD-associated trait rather than a state-dependent correlate of symptomatology.

In a double-blinded study with 41 OCD patients, Carmi et al. (32) randomly assigned patients to high-frequency (20-hz), low-frequency (1-hz), or sham deep transcranial magnetic stimulation. The researchers examined the theta band at the Cz electrode during a Stroop task and found treatment-related reductions in ERN following treatment. Replication is needed, but this suggests that flanker and Stroop error-related activity differ, and that the latter may change with treatment.

## Cognitive-Related Potentials

Another relevant ERP component is the P300: a positive voltage waveform observed ~300 ms after a low-probability (oddball) target or novel stimulus. It is a correlate of attention allocation and working memory while one is processing new or salient information [reviewed in (33, 34)]. The P300 is thought to arise from a widely distributed brain network including the bilateral medial frontal gyrus, the supramarginal gyri, the anterior cingulate cortex, and the orbitofrontal cortex (35, 36). These regions overlap with those associated with OCD pathophysiology (37, 38).

The P300 is commonly elicited using an auditory oddball paradigm (39). In this task, repetitive sounds are infrequently interrupted by a variant sound to which the participant must respond. Studies employing this paradigm before and after OCD treatment have found that P300 amplitude and frequency differ at baseline in patients relative to controls, but that only the amplitude may show changes post treatment. At baseline subjects with untreated OCD showed reduced P300 amplitudes and longer latencies relative to healthy controls (40). Given that EEG signals are elicited from summated neural activity, a lower P300 amplitude coupled with a longer latency (response delay) may indicate that while neurons are still firing, they are less synchronized in OCD patients. Following SSRI treatment, P300 normalized, but latency did not change. Higher P300 amplitudes were correlated with reductions in the YBOCS. The dissociation of P300 amplitude and latency suggests that they reflect distinct processes. Similar results have been reported 1-year post psychotherapy and pharmacology trial: Post-treatment assessment showed increased P300 amplitude, closer to that seen in controls (41). This increase strongly correlated with reductions on the YBOCS, with no change in P300 latency.

These reports contrast with several studies that have not found reduced baseline P300 in OCD (42, 43). Indeed, in one treatment study, individuals with OCD had increased P300 amplitude at baseline compared to healthy controls (35). Following semi-standardized psychotherapy and psychopharmacological treatment (sertraline; 50–150 mg), P300 amplitude in the oddball paradigm declined. No changes in latency were observed. P300 amplitude at baseline in OCD may vary depending on technical factors or on the specific population studied but normalize with treatment. Thus, more work is needed to characterize the relationship of the P300 to OCD treatment response.

## Oscillatory Markers

EEG power in specific frequency bands may be useful as a correlate of treatment response. A single study by Figee et al. (44) reported EEG oscillations after symptom provocation were strongly associated with therapeutic deep brain stimulation (DBS). In this study, 16 participants with OCD underwent nucleus accumbens-frontal network targeted DBS and showed stable clinical improvements for at least 1 year (44). DBS attenuated an increase in low-frequency activity seen after presentation of symptom-provoking stimuli. These EEG findings were complemented by a simultaneous fMRI analysis, highlighting the strength of a multi-modal imaging approach. Such multimodal investigations, combining EEG with fMRI, or other forms of imaging, are sparse in the OCD literature.

## EEG Complexity

The literature examining EEG correlates and predictors of OCD treatment outcome has predominantly focused on ERPs or individual oscillations (45). However, EEG signals comprise complex nonlinear interactions across space, time, and frequency bands; examining individual waveforms or locations misses much of this complexity. Newer analytic techniques that consider these nonlinear dynamics have recently been developed and applied in studies of schizophrenia, psychosis, Alzheimer's, seizure, and more recently, OCD (46, 47).

One complexity measure is approximate entropy (ApEn). ApEn is the quantification of how unpredictable a pattern of fluctuations is in a time series (48, 49). A high ApEn value indicates a more random system; a low value indicates a system with more predictable patterns. In one study, Altuglu et al. recruited 57 OCD patients with average YBOCS scores in their mid-20s, half of whom were treatment-resistant and half of whom were treatment-responsive. Treatment resistance was defined stringently (failure to improve on the YBOCS after an adequate trial of SSRIs and CBT). ApEn was examined across frequency bands in treatment-resistant and treatment-responsive patients. The authors found that ApEn complexity values extracted from the beta band specifically discriminated best between groups: There was lower complexity in the treatment-resistant group across the whole brain. There was a statistically significant inverse correlation (*r* = −0.21 to *r* = −0.33) between beta band complexity and YBOCS scores across frontal, parietal, and occipital channels.

Another study examined whether complexity of EEG-arousal regulation at rest could predict treatment response (50). Participants underwent a 15-min resting-state EEG and were then randomized to 3–6 months of psychotherapy, pharmacology, or a combination. A repeat EEG session was conducted following treatment. When comparing treatment responders to non-responders, responders had less complex neural patterns at baseline and spent significantly less time at the highest CNS arousal stage. This finding was particularly pronounced in those who had undergone the combination intervention.

## Source Localization in EEG Studies of OCD Treatment

A notable limitation of EEG has been the difficulty of identifying where in the brain the measured oscillatory signals arise. Although all EEG outputs are measured at the scalp in two dimensions, they are generated in the underlying three-dimensional brain. It is difficult to determine where in the brain the observed electrophysiological activity originates (51). EEG's poor spatial resolution is attributable to several factors, including head and/or scalp modeling errors, as well as EEG noise that can limit source localization calculation accuracy (52). Recently, a mathematical strategy to address this limitation has emerged: Low-Resolution Brain Electromagnetic Tomography (LORETA); see (53, 54). LORETA uses signals measured at surface electrodes to infer the distribution of current source density through the full brain volume (55). Importantly, LORETA has relatively low spatial resolution—typically, the brain is segmented into 2,394 voxels. This contrasts to the higher resolution—tens of thousands of voxels—of modern MRI imaging. Thus, LORETA's source localization is not as reliable a model of regional brain activity as fMRI, and its use has been controversial in some fields. Nevertheless, LORETA has recently been applied to several DSM-5 diagnostic categories, including OCD.

Using resting-state EEG, Krause et al. (56) used LORETA in a prospective design to characterize treatment response in OCD patients undergoing 10 weeks of concurrent psychotherapy and pharmacotherapy. Participants were categorized as treatment responders or non-responders based on reported YBOCS symptom reduction. At baseline, responders had significantly lower power in the beta 1 (12.5–18 Hz), beta 2 (18.5–21.0 Hz), and beta 3 (21.5–30.0 Hz) bands, as well as reduced activity in alpha 2 (10.5–12.0 Hz), localized to the anterior cingulate cortex. At follow-up, when compared to baseline, responders showed lower resting-state activity in beta 1 and 3 bands, as well as the alpha 2 band localized in the orbito-frontal cortex. The opposite pattern was seen in non-responders, reinforcing this association. In another study examining resting-state EEG before and after pharmacological treatment, lower pre-treatment activity in the beta band within the rostral anterior cingulate and medial frontal gyrus was associated with greater therapeutic response (2). Together, these studies suggest that beta power in the anterior cingulate is a candidate predictor of treatment response in OCD. However, the literature is sparse, and more work is needed.

## Discussion

We provided a brief narrative summary of studies examining EEG in relation to treatment outcome in OCD. The included studies are summarized in Table 1.

**Table 1:** Study summary.

**Article**	**Participants**	**Treatment type**	**Region/bands of focus**	**Task type**	**Primary analysis method**	**Framework**	**Primary finding**
Andreou et al. (35)	OCD: *n* = 76, control: *n* = 71	Behavior therapy and SSRI	32 channels (29 channel cap + 3 referenced to Cz)	Auditory oddball. eyes closed.	Two-tailed *t*-tests for independent samples; Spearman's rank correlation coefficient	Correlation	Increased activity for OCD patients in networks implicated with P300. Reduced with treatment.
Carmi et al. (32)	OCD: *N* = 41, HF: *n* = 16, LF: *n* = 8, Sham: *n* = 14	Deep TMS: High-frequency (20 Hz), low-frequency (1 Hz), sham	Cz, Theta Band	Stroop	Mixed ANOVA	Correlation	Treatment-related reductions found in ERN following Deep TMS
Dohrmann et al. (50)	*N* = 51, 30 F	CBT and pharmacotherapy	31 electrodes (Fp1, 2, 3, 4, 7, 8, z/Fc1, 2, 5, 6/C3, 4 z/FT9, 10/T7, 8/CP5, 6/TP9, 10/P3, 4, 7, 8, z/O1, 2/PO9, 10)	Resting state, 15 min	Multi-variate analysis of covariance (MANCOVA)	Prediction	CNS arousal markers discriminates between OCD treatment responders and non-responders.
Figee et al. (44)	OCD *=* 16, control *=* 13	Deep brain stimulation	International 10/10 system with 64 electrodes	Symptom provocation	Repeated measures ANOVA	Correlation	DBS attenuated the brain's frontal response to symptom provoking stimuli
Fontenelle et al. (57)	OCD: *n* = 17, (responder = 10, non-responder = 7)	12 weeks + of medication, primarily SRIs, non-SRI tricyclics, other medications prescribed for individual patient needs	International 10/20 System with earlobes as reference	Resting State	SPM-99 *t*-test for independent samples	Correlation	Lower pretreatment beta band activity in the rostral anterior cingulate and medial frontal gyrus associated with increased treatment response
Krause et al. (56)	*N* = 41, 18 F (OCD sample)	10 weeks combination CBT and SSRI (sertraline)	International 10/20 system with Cz as reference and Fpz as ground	Resting state	Linear and robust regression	Prediction	LORETA indicated that brain activity increased in responders and decreased in nonresponders
Riesel et al. (30)	OCD: *n* = 45, 22 F; control: *n* = 39, 221 F	30 CBT sessions, some medicated	64 electrodes, Cz as reference	Flanker Task	Repeated-measures Analysis of Variance (ANOVA)	Correlation	Pretreatment differences between OCD patients and healthy controls showed stable error-related and correct-related negativity following treatment.
Sanz et al. (40)	OCD: *n* = 19, 10 F; control: *n* =19, 9 F	Clomipramine (250–300-mg)	International 10–20 system including Pz; 20 tin electrodes inserted in pre-configured cap	Auditory Oddball	Independent Samples *t*-test	Correlation	P300 varied between healthy controls and treatment-free OCD participants. Increase in P300 after treatment
Yamamuro et al. (41)	OCD: *n* = 14; control: *n* = 10	1 year of psychotherapy and pharmacotherapy	Fz, Cz, C3, C4, and Pz	Auditory oddball	Two-tailed paired *t*-test; Spearman's correlation coefficient	Prediction	Pharmacotherapy and psychotherapy improved P300 after 1 year of treatment

*HF, High-frequency; LF, Low-frequency; CBT, Cognitive-behavioral therapy; SSRI, Selective Serotonin Reuptake Inhibitor; DBS, Deep brain stimulation; TMS, Transcranial magnetic stimulation*.

The most striking conclusion from this brief review is how limited this literature is. Given the convenience and cost of EEG relative to MRI or PET imaging—and the consequent feasibility of deploying EEG measures at scale in clinical settings—such investigations merit closer attention.

An asymmetry uncovered by this review was between studies employing predictive vs. correlational methods. Few studies have attempted to truly predict behavior from EEG features (41, 50, 56), instead reporting descriptive associations between EEG features and clinical change. While this problem is not unique to the EEG literature (15), it is imperative for researchers to distinguish between studies that make causal or predictive claims vs. those that report correlations with symptom change. Larger, prospectively designed and cross validated studies are critical to better conceptualize the relationships between EEG measurements and OCD-related outcome variables (16).

Despite the thinness of this literature, there are clearly several avenues for future research. Notably, ERPs remain underexplored. The directionality, uniformity, and magnitude of change following treatment interventions remains unclear for the P300 and ERN/CRN. These discrepancies may be attributable to small sample sizes or differences in participant characteristics (e.g., severity, medication status, treatment type). For example, Yamamuro et al. (41), in a small sample (*N* = 14), found lower P300 amplitude at Cz and C4 at baseline in OCD; but this has not been consistently corroborated by other studies. Sanz et al. (40) also found lower P300 amplitude at baseline but found this at the Pz, not the Cz, and C4. Both studies found a statistically significant decrease in the P300 following pharmacotherapy and/or psychotherapy. This suggests that change in the P300 may be associated with symptom improvement with treatment, but research is needed to clarify these effects.

Another important variable in these studies is treatment type. Although most studies in this review combined pharmacotherapy and psychotherapy, their individual impact on brain function and their differential benefit to certain subsets of patients remains unknown. Sanz et al. (40) emphasize the role of the serotonergic system's influence on OCD pathophysiology, and by implication on EEG abnormalities associated with the condition, but their data cannot directly establish this. No studies to date have used EEG to examine the effects of CBT in unmedicated OCD or to systematically compared CBT to pharmacotherapy. Recent fMRI literature suggests that functional connectivity between large-scale brain networks changes following CBT (58); it will be fruitful to use EEG measures, which probe different aspects of brain network organization than fMRI, to address similar questions in homogenous patient samples.

Recent advances in EEG data processing are allowing for more complex and efficient analyses and better source localization. For example, Dohrmann et al. (50) used arousal regulation and CNS wakefulness stages to predict OCD treatment response. Fontenelle et al. (57) localized lower beta band activity in OCD to the rostral anterior cingulate and medial frontal gyrus, while Krause et al. (56) found differential beta band effects in treatment responders compared to nonresponders. These analytic approaches have the potential to provide a clearer picture of brain correlates of treatment reponse in OCD at the level of regions, networks, and frequency patterns.

## Conclusion

PET and fMRI have several advantages, including their ability to identify areas of interest with high spatial resolution (9). Although EEG has lower spatial resolution, it measures qualitatively different characteristics of brain function, including oscillatory organization, and has a temporal resolution measured in milliseconds (20). Further, EEG may have more practical potential for widespread clinical deployment. As such, identifying actionable associations with treatment outcome is critical. Recent advances, such as techniques for band-specific source localization will only increase the potential of EEG analyses in the coming years. Overall, the literature examining associations between EEG measures of brain organization and OCD treatment outcomes is sparse, and more research is needed.

## Author Contributions

BZ: responsible for half of the manuscript writing, the manuscript conceptualization, organization, and synthesis of EEG literature with OCD treatment. Expert on CBT for OCD. ES: responsible for literature review, other half of manuscript writing, and managing editorial process. PS: expert consultant on EEG research and methods, provided conceptual, organizational suggestions for the paper, and provided editorial suggestions on technical aspects of EEG. CP: provided primary editorial suggestions on entire paper, served as expert consultant on neurobiology, and psychiatric treatment of OCD. All authors contributed to the article and approved the submitted version.

## Author Disclaimer

The views and opinions expressed are those of the authors.

## Conflict of Interest

The authors declare that the research was conducted in the absence of any commercial or financial relationships that could be construed as a potential conflict of interest.

## Publisher's Note

All claims expressed in this article are solely those of the authors and do not necessarily represent those of their affiliated organizations, or those of the publisher, the editors and the reviewers. Any product that may be evaluated in this article, or claim that may be made by its manufacturer, is not guaranteed or endorsed by the publisher.
